# Quantification and epigenetic evaluation of the residual pool of hepatitis B covalently closed circular DNA in long-term nucleoside analogue-treated patients

**DOI:** 10.1038/s41598-020-78001-1

**Published:** 2020-12-03

**Authors:** Fanny Lebossé, Aurore Inchauspé, Maëlle Locatelli, Clothilde Miaglia, Audrey Diederichs, Judith Fresquet, Fleur Chapus, Kamal Hamed, Barbara Testoni, Fabien Zoulim

**Affiliations:** 1 INSERM U1052-Cancer Research Center of Lyon (CRCL), Lyon, France; 2 University of Lyon, UMR_S1052, CRCL, Lyon, France; 3 Department of Hepatology, Croix Rousse Hospital, Hospices Civils de Lyon, Lyon, France; 4 Novartis Pharmaceuticals Corporation, East Hanover, NJ USA

**Keywords:** Hepatitis B virus, Hepatitis B, Hepatitis B

## Abstract

Hepatitis B virus (HBV) covalently closed circular (ccc)DNA is the key genomic form responsible for viral persistence and virological relapse after treatment withdrawal. The assessment of residual intrahepatic cccDNA levels and activity after long-term nucleos(t)ide analogues therapy still represents a technical challenge. Quantitative (q)PCR, rolling circle amplification (RCA) and droplet digital (dd)PCR assays were used to quantify residual intrahepatic cccDNA in liver biopsies from 56 chronically HBV infected patients after 3 to 5 years of telbivudine treatment. Activity of residual cccDNA was evaluated by quantifying 3.5 kB HBV RNA (preC/pgRNA) and by assessing cccDNA-associated histone tails post-transcriptional modifications (PTMs) by micro-chromatin immunoprecipitation. Long-term telbivudine treatment resulted in serum HBV DNA suppression, with most of the patients reaching undetectable levels. Despite 38 out of 56 patients had undetectable cccDNA when assessed by qPCR, RCA and ddPCR assays detected cccDNA in all-but-one negative samples. Low preC/pgRNA level in telbivudine-treated samples was associated with enrichment for cccDNA histone PTMs related to repressed transcription. No difference in cccDNA levels was found according to serum viral markers evolution. This panel of cccDNA evaluation techniques should provide an added value for the new proof-of-concept clinical trials aiming at a functional cure of chronic hepatitis B.

## Introduction

Almost 257 million people worldwide are chronically infected with hepatitis B virus (HBV) and consequently predisposed to an increased risk of cirrhosis and hepatocellular carcinoma (HCC)^1,2^. The hallmark of HBV infection is the presence of covalently closed circular DNA (cccDNA) in the nucleus of infected hepatocytes^3^.

cccDNA is a stable episomal viral “minichromosome” and serves as the template for the transcription of all viral RNAs, amongst which is the pre-genomic (pg)RNA, specifically transcribed from cccDNA and not from integrated viral sequences. pgRNA is retrotranscribed by the viral polymerase into relaxed circular DNA (rcDNA) within the newly formed nucleocapsids in the cytoplasm^3,4^. The intrahepatic cccDNA pool can be maintained by both intracellular recycling of nucleocapsids or by new rounds of infection and accounts for : (i) the chronicity of HBV infection, (ii) viral relapse following treatment withdrawal and (iii) for viral reactivation under immunosuppressive conditions for patients with cured HBV infection^5,6^. cccDNA is decorated by nucleosomes to form an organized chromatin structure, a so-called viral “minichromosome”, which is epigenetically regulated by host and viral factors (reviewed in^7^). Acetylation of cccDNA-associated histone tails has been correlated in vivo to patients’ viremia levels and intrahepatic cccDNA transcriptional activity^8,9^, while the association of histone H3 trimethylation of lysine 9 and 27 with viral transcription levels in vivo is more controversial^9,10^.

Nucleos(t)ides analogues (NUCs), the standard-of-care treatment for chronic hepatitis B (CHB), suppress serum viral DNA, but do not have a direct effect on nuclear cccDNA^4–6,11,12^. Even if recent data suggest that NUC therapy is unable to completely block intracellular viral DNA synthesis, which may contribute to the continuous replenishment and, thus, to the inability to clear the cccDNA pool^13^, long term therapies decrease intrahepatic cccDNA levels, challenging the sensitivity of current cccDNA quantification techniques. Indeed, in a recent study, Lai et al*.* failed to detect any intrahepatic cccDNA for half of the 43 patients analyzed after 10 years of NUC therapy, whereas some of the cccDNA-negative samples were positive for pgRNA and total intrahepatic HBV DNA (tHBV DNA)^14^. However, it has to be noted that the tHBV DNA assay used in that study spans the HBVS open reading frame, and thus does not exclude amplification of viral integrated sequences in the host genome.

The research priorities to achieve a cure of chronic hepatitis B were recently summarized by the International Coalition to Eliminate HBV (ICE-HBV) consortium and include a better characterization of correlates of cure to help the evaluation of novel drugs in clinical trials^15^. The evaluation of cccDNA is thus paramount but still faces several challenges with the need of: (i) more sensitive techniques for cccDNA quantification to overcome the challenges of small size liver samples obtained from liver biopsies and also of low levels of intrahepatic viral DNA during therapy; (ii) coupling cccDNA quantification and cccDNA transcriptional activity evaluation, the latter being appraised by its epigenetic status and pgRNA levels; (iii) investigating the evolution of the number of infected hepatocytes harboring cccDNA in the liver; (iv) developing in-situ, single-cell, single-molecule assays to visualize cccDNA in infected cells.

Here, we report the use of a panel of investigational molecular biology assays to robustly profile HBV replicative markers, in particular levels of cccDNA and its transcriptional activity, in liver biopsies of long-term NUC treated patients. Extensive evaluation by different techniques, including a highly sensitive droplet digital PCR (ddPCR) method and a liver biopsy—adapted chromatin immunoprecipitation (ChIP)-quantitative PCR technique (micro-ChIP), allowed to reveal the presence of detectable cccDNA in all except one patient out of a cohort of 56 patients treated for 3 to 5 years with the nucleoside analogue telbivudine. Moreover, the residual pool of cccDNA was associated with histone post-transcriptional modifications (PTMs) related to repressed transcription.

## Results

### Patients’ characteristics

At the time of enrolment in the trial (Supplementary Fig. 1), all 56 patients (37 HBeAg( +) and 19 HBeAg(−)) had alanine aminotransferase (ALT) levels higher than the upper limit of normal (ULN) and were classified as chronic hepatitis B (CHB) according to the 2017 EASL clinical practice guidelines^16^. The median age was 29 years old, HBV e antigen-positive (HBeAg( +)) patients being significantly younger than HBV e antigen-negative (HBeAg(−)) ones. Forty-three patients were infected with HBV genotype C and 13 with genotype B (Table 1). Baseline serum HBV DNA and ALT levels tended to be higher for HBeAg( +) patients and there was no significant difference between the two groups in the duration of telbivudine treatment (Table 1). Before treatment, the majority of the patients exhibited low histological scores of necroinflammatory activity and fibrosis although HBeAg(−) group showed a significantly higher proportion of patients with advanced fibrosis (Supplementary Fig. 2).

**Table 1:** Demographical and virological patients’ characteristics at baseline.

	Total cohort (n=56)	Baseline HBeAg(+) patients (n=37)	Baseline HBeAg(–) patients (n=19)	p value
Age^a,b^ (years)	29 (25–41)	27 (23–33)	39 (30–44)	0.0004
**Viral genotype**^c^				
B C	13 43	11 26	2 17	ns
Baseline serum HBV DNA^a,b^ (IU/mL)	1.2 × 10^8^ (1.4 × 10^6^–7 × 10^8^)	2.1 × 10^8^ (2.7 × 10^7^–1.2 × 10^9^)	2.2 × 10^6^ (2.8 × 10^5^–5.4 × 10^8^)	ns
Baseline ALT level^a,b^ (IU/L)	1.6 × 10^2^ (9.4 × 10^1^–2.6 × 10^2^)	1.6 × 10^2^ (1 × 10^2^–2.5 × 10^2^)	1.3 × 10^2^ (6.2 × 10^1^ 3.4 × 10^2^)	ns
Duration of telbivudine treatment^a,b^ (weeks)	234 (157–261)	208 (157–261)	260 (158–261)	ns

^a^Data are expressed as median (1st quartile–3^rd^ quartile)

^b^Mann Whitney U test was used for comparison between HBeAg( +) and (−) groups.

^c^χ^2^ test was performed between HBeAg( +) and (−) groups.

At the time of liver biopsy, i.e. one week after entry in the CLDT600ACN04E1 study, corresponding to three to five years of telbivudine therapy (Supplementary Fig. 1 and Methods section), all the patients reached ALT levels under the ULN and only 3/56 patients exhibited a detectable serum HBV DNA (all 3 HBeAg( +) patients).

### Quantification of intrahepatic viral markers after telbivudine treatment using a qPCR method

**Figure 1:**
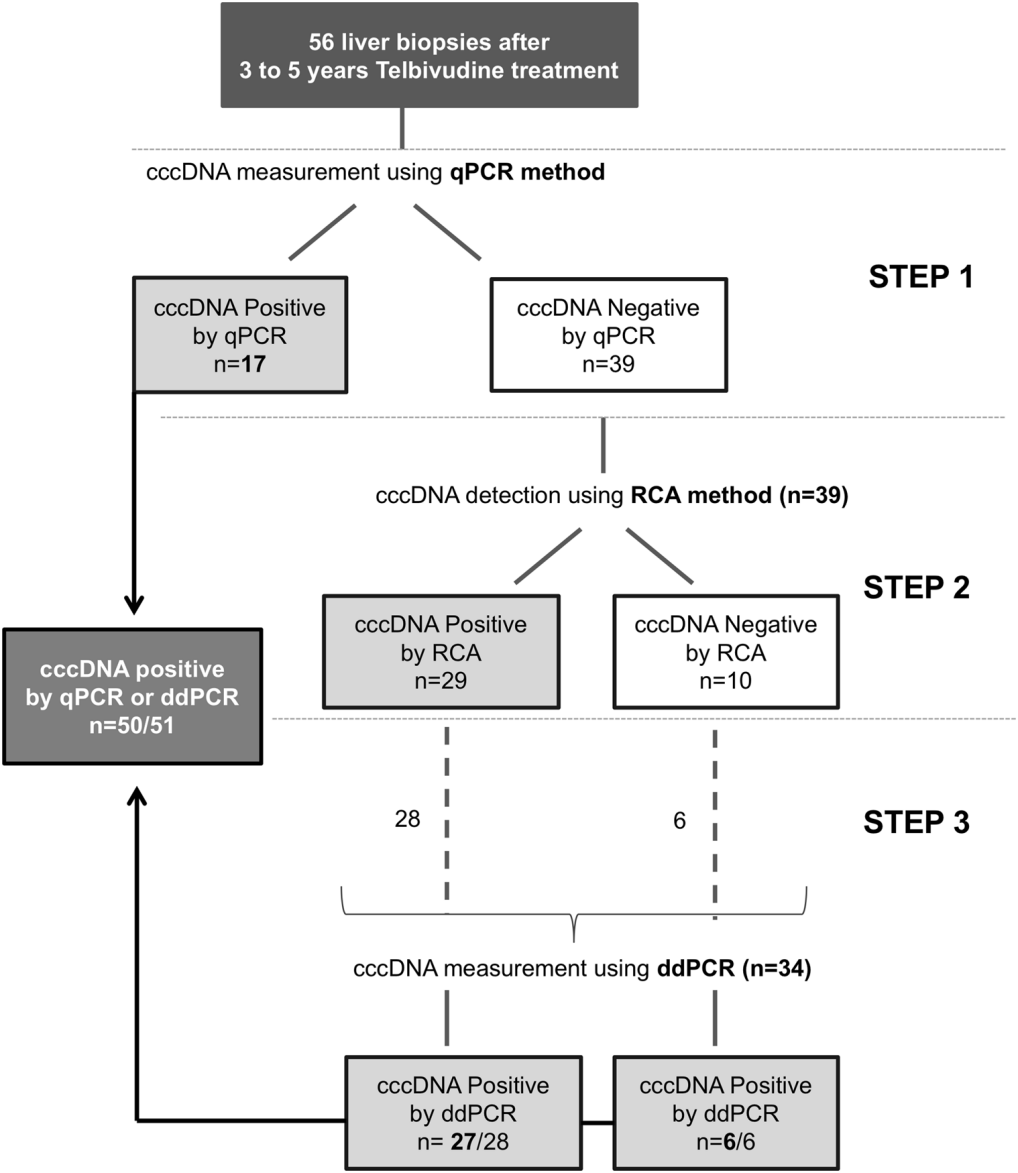
Workflow of intrahepatic covalently closed circular DNA (cccDNA) assessment. Step 1: Frozen liver biopsy samples were first tested for the presence and the level of intrahepatic cccDNA using quantitative PCR (qPCR) method^17^; Step 2: Rolling Circle Amplification (RCA)^35,36^ was performed on the 39 qPCR-negative samples and detected cccDNA in 29 of them; Step 3: digital droplet PCR (ddPCR) was performed on qPCR-negative liver samples with available remaining material (28 out of 29 RCA-positive samples and 6 out of 10 RCA-negative samples). ddPCR detected cccDNA for all-but-one samples tested. Overall, intrahepatic cccDNA was detectable in 50 out 51 patients tested when assessed by qPCR or ddPCR. *cccDNA* covalently closed circular DNA, *RCA* rolling circle amplification, *ddPCR* droplet digital PCR.

Intrahepatic total HBV DNA (tHBV-DNA), cccDNA and 3.5 kb RNA (preC/pgRNA) were first quantified using a qPCR method already described for intrahepatic HBV markers quantification^17^ and results are listed in Table 2. tHBV-DNA levels after telbivudine therapy were less than 1 copy per cell in all patients. Intrahepatic cccDNA level was under the limit of detection for 39 out of 56 (70%) patients (Fig. 1). Baseline HBeAg( +) patients showed a significantly higher proportion of patients with detectable cccDNA (15 out of 37 HBeAg( +) patients) than HBeAg(−) patients, where only 2 out of 19 patients had detectable cccDNA (Table 2). Similar to tHBV-DNA, intrahepatic low levels of 3.5 kb RNA were detected after telbivudine treatment (median of 1.98 × 10^–1^ copies per cell, 2.08 × 10^–1^ copies per cell and 1.98 × 10^–1^ copies per cell, for the entire cohort, HBeAg( +) and HBeAg(–) patients, respectively (Table 2)).

**Table 2:** Patients’ outcomes at the time of liver biopsy.

		Total cohort (n=56)	Baseline HBeAg(+) patients (n=37)	Baseline HBeAg(–) patients (n=19)	p value
**Serum**	**Virological outcomes** HBe Ag loss HBe seroconversion Anti-HBe antibodies HBs Ag loss HBs seroconversion	28 15 30 2 0	28 15 15 2 0	– – 15 0 0	
	**Serum HBV DNA**^**a,b**^ (detectable/undetectable/missing value)	3/50 /3	3/32/2	0/18/1	ns
	**ALT level**^**c,d**^ (IU/L)	24 (18–38)	23 (18–31)	34 (19–48)	ns
**Liver**	**Intrahepatic total HBV DNA**^**c,d,e**^ (copies/cell)	1.49 × 10^−^^1^ (5.23 × 10^−^^2^–2.61 × 10^−^^1^)	1.76 × 10^−^^1^ (8.9 × 10^−^^2^–2.76 × 10^−^^1^)	7.7 × 10^−^^2^ (4.4 × 10^−^^2^ – 2.43 × 10^−^^1^)	ns
	**Intrahepatic cccDNA**^**b,e**^ (Detectable/undetectable)	17/39	15/22	2/17	0.002
	**Intrahepatic 3.5 kb RNA**^**c,d,e**^ (Relative quantity)	1.98 × 10^−^^1^ (6.20 × 10^−^^2^–6.03 × 10^−^^1^)	2.08 × 10^−^^1^ (8.18 × 10^−^^2^–6.44 × 10^−^^1^)	1.98 × 10^−^^1^ (5.10 × 10^−^^2^ – 5.74 × 10^−^^1^)	ns

^a^Data are available for 53 patients.

^b^χ^2^ test between HBeAg( +) and (−) groups.

^c^Data are expressed as median (1st quartile – 3rd quartile).

^d^Mann Whitney U test was used for comparison between HBeAg( +) and (−) groups.

^e^Data for intrahepatic viral DNA and RNA were obtained using the qPCR quantification method (See Patients and Methods section for details).

**Figure 2:**
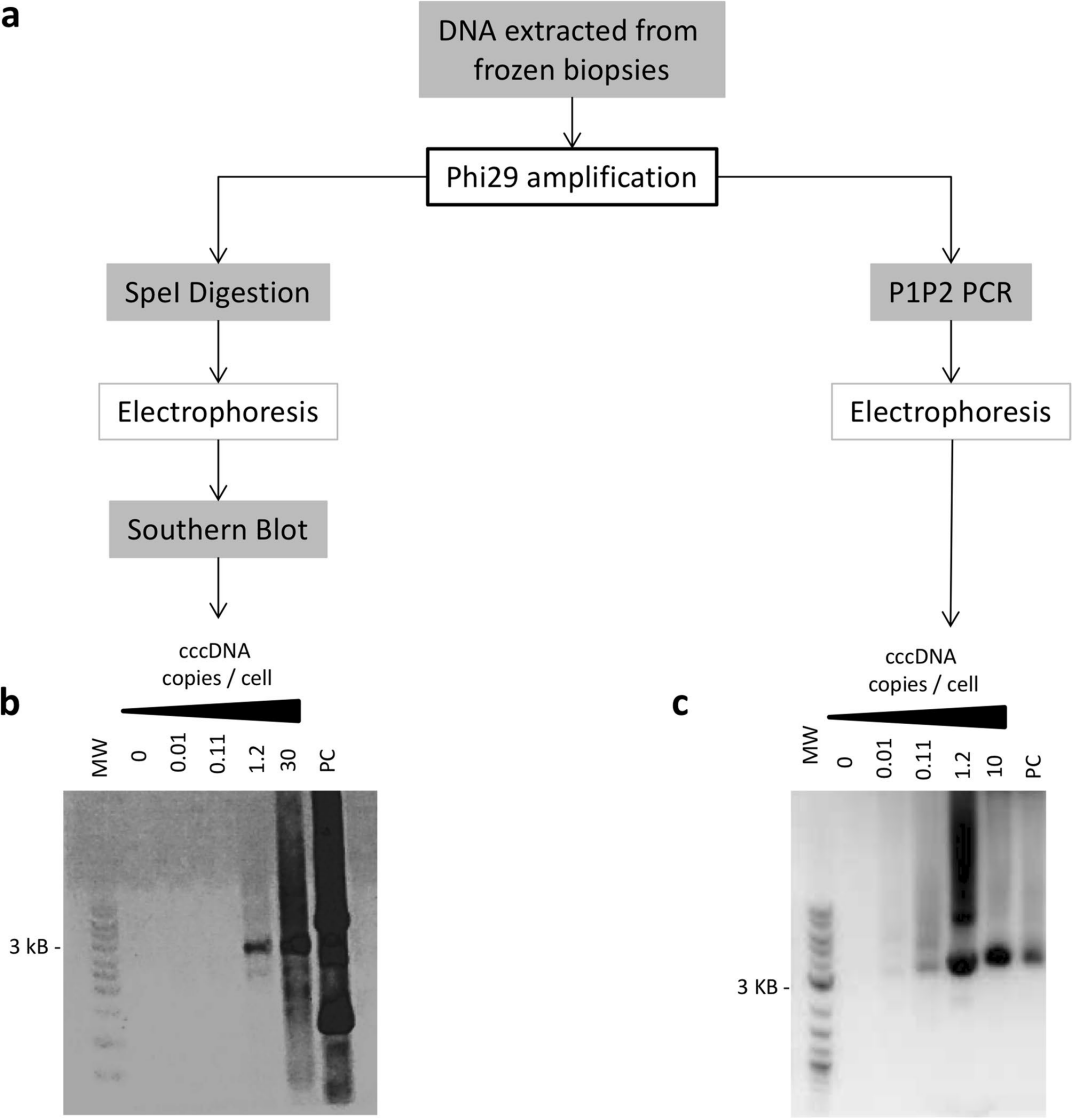
Rolling Circle Amplification (RCA) analysis on patients’ liver biopsies. **(a)** Workflow of RCA set-up in liver biopsies: DNA extracted from frozen-liver biopsies was first amplified with Phi29 polymerase for 21 h at 30 °C. Amplification products were then either digested with SpeI enzyme and analyzed according to Southern Blot technique using HBV-specific cold probes or assessed following a full-length HBV genomic PCR (P1-P2) followed by gel electrophoresis. **(b)** Examples of Southern Blot following RCA and SpeI digestion on liver biopsies from patients with different cccDNA concentration measured by qPCR and negative (0; H20) and positive controls (PC; plasmid containing a full-length HBV genome). **(c)** Examples of gel electrophoresis following full-length HBV genomic PCR (P1-P2) performed on RCA products from patients’ liver biopsies with different cccDNA concentration measured by qPCR and negative (0; H20) and positive control (PC; plasmid containing a full-length HBV genome). *MW* molecular weight; 0: negative control (H20); *PC* positive control (plasmid containing a full-length HBV genome).

### Intrahepatic cccDNA detection using rolling circle amplification and droplet digital PCR

**Figure 3:**
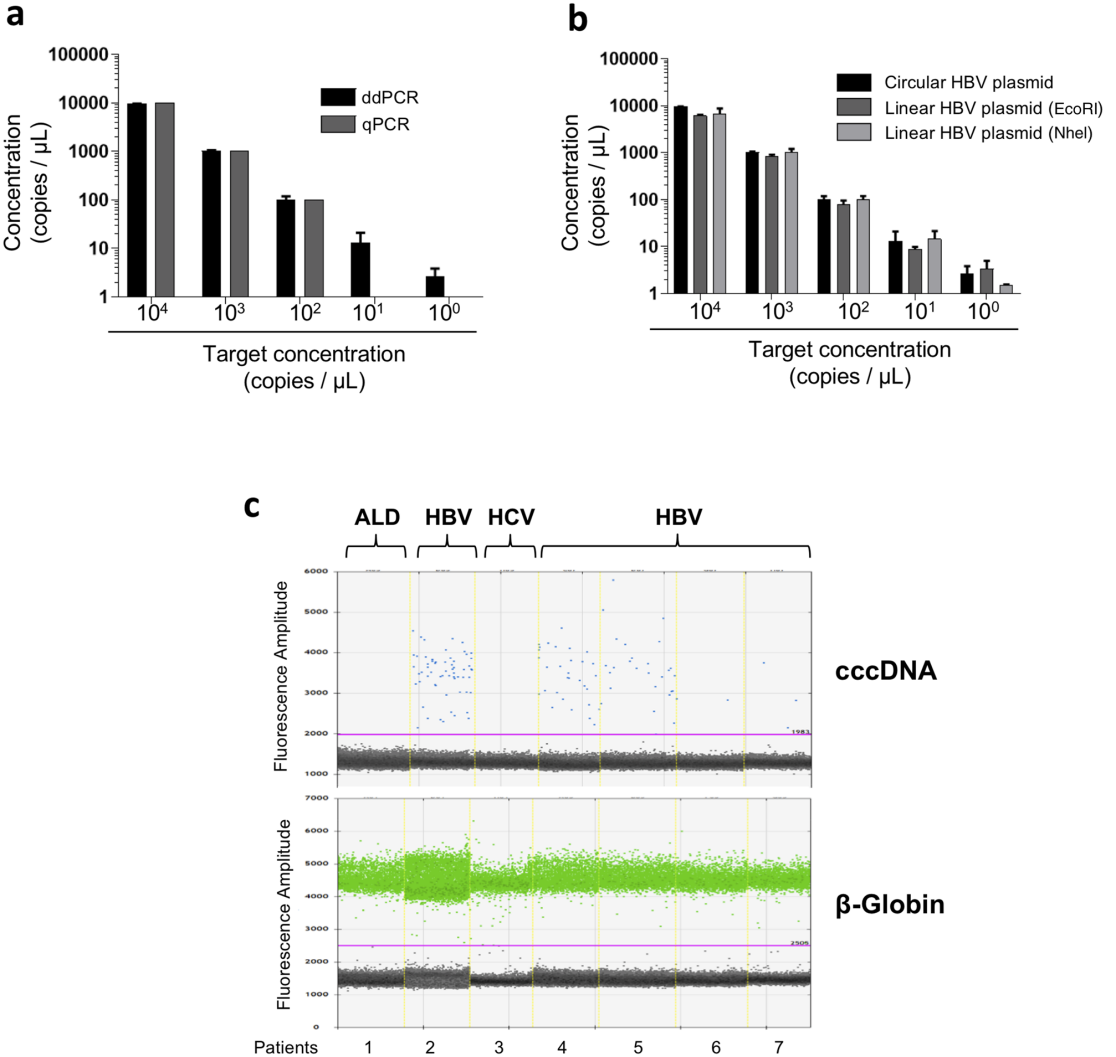
Comparison of droplet digital PCR (ddPCR) and qPCR methods for cccDNA quantification.** (a)** Serial dilutions of a known quantity of an HBV genome-containing plasmid (pBR322) were quantified by ddPCR (black bars) and qPCR (grey bars); **(b)** Serial dilutions of a known quantity of an HBV genome-containing plasmid (pBR322) were quantified by ddPCR before (black bars) or after linearization with two different restriction enzymes (dark and light grey bars). **(c)** 1-Dimension ddPCR analysis of cccDNA (upper panel) and β-globin (lower panel) quantification in liver tissue coming from HBV-positive (n = 5) and HBV-negative controls (n = 2; (alcohol-related liver disease, ALD, n = 1 and HCV chronic hepatitis (HCV, n = 1)). Each column, limited by yellow dotted lines, represents a sample. Each dot represents a droplet. Black dots = negative droplets; blue dots = droplets positive for cccDNA detection; green dots = positive droplets for β-globin detection. Violet line = background threshold. ALD: alcohol-related liver disease, HCV: hepatitis C virus.

To further investigate the presence of cccDNA in the liver samples with undetectable cccDNA by qPCR, we decided to perform two supplementary approaches: RCA and ddPCR (Fig. 1). Among the 39 samples with undetectable cccDNA by qPCR, 29 showed a positive signal after RCA analysis (Figs. 1 and 2). Since the major limitation of RCA technique is that it is not quantitative, we decided to use ddPCR, which has been recently shown to be more sensitive than qPCR in detecting HBV genome in low-level HBV infected samples^18–20^. We showed that the ddPCR cccDNA assay specifically amplifies episomal HBV DNA over integrated sequences and preferentially recognizes cccDNA over rcDNA (Methods section and Supplementary Fig. 3). In particular, we could confirm that ddPCR is 2 logs more sensitive than qPCR for the quantification of an HBV plasmid, allowing HBV DNA quantification from samples containing as less as 1 copy/µL of HBV genome (Fig. 3a). We also showed that the circular nature of cccDNA did not affect ddPCR efficiency, since the same quantification results were obtained from of a circular and a linearized HBV-plasmid template (Fig. 3b). The analysis of HBV-negative liver biopsy samples confirmed the specificity of the technique and served as reference for the settings of thresholds discriminating positive from negative droplets (Fig. 3c).

**Figure 4:**
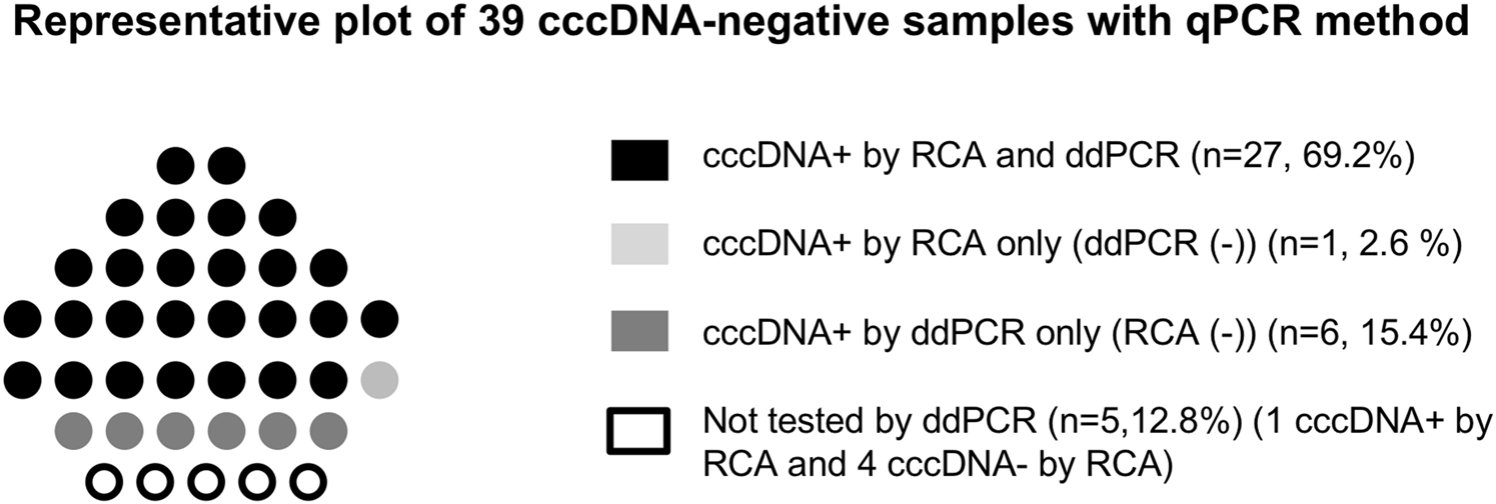
Contribution of rolling circle amplification (RCA) and droplet digital PCR (ddPCR) as additional methods to detect covalently closed circular DNA (cccDNA) on qPCR-negative liver samples. Presence of cccDNA was assessed on 39 qPCR-negative liver samples using either a RCA detection method or ddPCR quantification method. The presence of intrahepatic cccDNA was revealed by RCA and then confirmed and quantified by ddPCR for 27/39 patients (69.2%) (black dots). The ddPCR technique allowed the detection of cccDNA in 6 (15.4%) additional samples (RCA-negative; dark grey dots) whilst one sample (2.6%) was positive with RCA only (ddPCR-negative; light grey dots). Due to the insufficient remaining DNA material, 5 samples (12.8%) were tested for the presence of intrahepatic cccDNA by RCA only, without quantification with ddPCR technique (1 RCA-positive sample and 4 RCA-negative samples) (white dots). cccDNA: covalently closed circular DNA, RCA: rolling circle amplification, ddPCR; droplet digital PCR.

cccDNA-positive liver samples with qPCR were confirmed to be positive also by ddPCR. Twenty-seven out of 34 samples with undetectable cccDNA by qPCR had a positive cccDNA signal with both RCA and ddPCR (Fig. 4). ddPCR allowed the detection of cccDNA in 6 more patients than RCA while one sample was positive only by RCA analysis (Fig. 4). Altogether, after combining qPCR and ddPCR results, intrahepatic cccDNA was detectable in 50 out of 51 telbivudine-treated patients tested with both techniques (5 liver samples were not assessed by ddPCR due to insufficient residual material) (Figs. 1 and 4).

Overall, absolute amounts of cccDNA quantified by ddPCR were 4.43 × 10^−3^copies/cell for the entire cohort and 7.27 × 10^–3^ and 3.72 × 10^–3^ copies/cell for baseline HBeAg( +) and HBeAg(–) patients, respectively (Table 3). The cccDNA levels measured by ddPCR were significantly lower in samples with undetectable cccDNA by qPCR (median of 3.2 × 10^–3^ copies/cell *vs* 1.9 × 10^–2^ copies/cell in samples negative and positive for cccDNA with qPCR, respectively) (Table 3).

**Table 3:** Intrahepatic HBV cccDNA quantification by droplet digital (dd)PCR^a^.

	Total cohort (n = 49)^b^	Baseline HBeAg( +) patients (n = 31)	Baseline HBeAg(−) patients (n = 18)	*p-value*^c^	cccDNA detectable with qPCR (n = 15)	cccDNA undetectable with qPCR (n = 34)	*p-value*^c^
cccDNA (copies/cell)	4.43 × 10^–3^ (2.37 × 10^–3^–1.62 × 10^–2^)	7.27 × 10^–3^ (3.00 × 10^–3^–1.95 × 10^–2^)	3.72 × 10^–3^ (1.59 × 10^–3^–9.17 × 10^–3^)	ns	1.93 × 10^–2^ (9.84 × 10^–3^ –3.15 × 10^–2^)	3.18 × 10^–3^ (1.59 × 10^–3^–7.52 × 10^–3^)	< 0.0001

^a^Data are expressed as median (1^st^ quartile – 3^rd^ quartile).

^b^ddPCR was run on 15/17 cccDNA-positive and 34/39 cccDNA-negative liver samples after qPCR analysis.

^c^Mann Whitney U test was used for comparison between groups.

At the time of liver biopsy, 28/37 (76%) HBeAg( +) patients had lost HBeAg and 15/37 (40%) had experienced HBe seroconversion (Table 2). No differences in baseline age, serum HBV DNA and ALT levels were found between patients who experienced or not HBeAg loss and/or seroconversion (Supplementary Table 1). No differences in intrahepatic tHBV-DNA, cccDNA or 3.5 kb RNA levels after telbivudine therapy were reported according to HBeAg loss and/or seroconversion at the time of liver biopsy (Supplementary Table 1 and 2). Two out of 3 patients with detectable serum HBV DNA at the time of liver biopsy showed positive cccDNA when assessed with qPCR, while all of them were positive when tested by ddPCR. No difference of tHBV-DNA or 3.5 kb RNA was found for these patients compared to those with undetectable serum HBV DNA (data not shown). Only two patients (HBeAg( +) at baseline) lost HBsAg after telbivudine therapy, therefore it was not possible to perform statistical analysis in this group of patients (Table 2). However, both had undetectable cccDNA by qPCR, but scored positive by ddPCR quantification.

### cccDNA epigenetic status on telbivudine treatment

**Figure 5:**
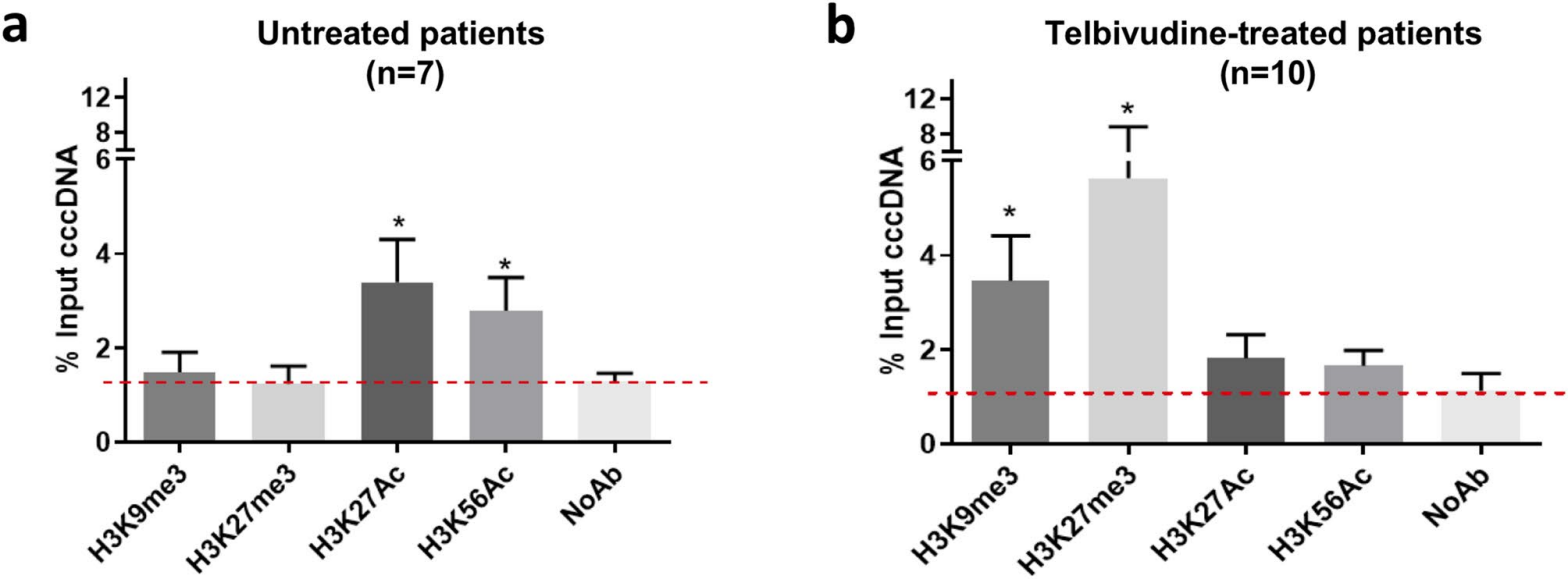
Covalently closed circular DNA (cccDNA) epigenetic analysis under long-term Telbivudine treatment. Chromatin Immunoprecipitation analysis was performed using specific antibodies against H3K9me3, H3K27me3, H3K27Ac and H3K56Ac. Signal enrichment is expressed as the percentage of input cccDNA in untreated chronic hepatitis B comparative group (n = 7) **(a)** and Telbivudine-treated patients (n = 10) **(b)**. Mann–Whitney U test was used to compare enrichment of specific antibodies *vs* the negative control (NoAb), alpha threshold = 0.05; * p < 0.05. *cccDNA* covalently closed circular DNA, *NoAb* negative control.

3.5 kb RNAs are exclusively transcribed from its cccDNA template^3,5^. Given the low levels of 3.5 kb RNAs found in telbivudine-treated patients, we wondered if it could be explained not only by a low level of its transcriptional template, i.e. cccDNA, but also by a reduction of its transcriptional activity. To test this hypothesis, we performed ChIP analysis on liver samples derived from 10 telbivudine-treated patients with sufficient liver material (4 HBeAg( +), 6 HBeAg(−)). All the patients had qPCR-detectable cccDNA, but did not differ significantly from the cccDNA-qPCR negative Telbivudine-treated patients not included in ChIP analysis, except for having higher cccDNA levels (Supplementary Table 3). They were compared to 7 untreated CHB patients with active HBV transcription and replication (5 HBeAg( +), 2 HBeAg(−)) (see Supplementary Table 4 for patients’ characteristics). We assessed the levels of cccDNA-associated histone PTMs associated to either active (H3K27Ac, H3K56Ac) or inactive (H3K9me3, H3K27me3) transcription, using GAPDH promoter amplification as a control for antibody specificity (Supplementary Fig. 4a)^21^. Untreated CHB patients presented a higher 3.5 Kb RNA/cccDNA ratio (median of 160 *vs* 6.03 in telbivudine-treated group, p = 0.0001, Supplementary Table 4) associated with a significant enrichment of positive histone PTMs on cccDNA (Fig. 5a), while telbivudine-treated patients showed no significant enrichment with respect to No Antibody control of positive histone PTMs, but a significant increase of histone PTMs associated with repressed transcriptional activity, independently from patients’ HBeAg status (Fig. 5b and Supplementary Fig. 4b-c).

## Discussion

In the context of the global HBV cure research programs^15^, the evaluation of the intrahepatic cccDNA amount and its transcriptional activity will be instrumental for the assessment of the efficacy of the novel antiviral strategies under clinical investigation and for the development of non-invasive biomarkers reflecting the pool of cccDNA^15^.

Here, we propose the use of a panel of molecular assays to robustly assess both cccDNA amount and transcriptional activity in vivo, in liver samples derived from long-term NUC-treated patients. The implementation of ddPCR, together with the adaptation of classic ChIP to small size liver biopsies represent an advent by providing technical tools to evaluate the residual pool of cccDNA and its epigenetic status in the infected liver.

Similar to the study of Lai et al*.*, we found that the majority of the patients had undetectable levels of intrahepatic cccDNA after long course of NUC treatment, when assessed with qPCR technique^14^. However, in our study, using a more sensitive ddPCR assay, we could detect and quantify intrahepatic cccDNA in all but one tested liver biopsies of telbivudine-treated patients. Importantly, the assay used in this study for cccDNA quantification specifically recognize episomal cccDNA over HBV integrated sequences (Supplementary Fig. 3). The relevance of ddPCR analysis in CHB has already been reported for the investigation of CHB patients who received interferon therapy^19^ and in patients with occult HBV infection^20^. Our results provide new information in long-term virally suppressed patients supporting the implementation of ddPCR technology for the evaluation of cccDNA with newer and more potent antivirals undergoing clinical trial evaluation. Our results are also consistent with previous mathematical modelling of cccDNA kinetics that showed low rates of clearance in NUC-treated patients^13^ and provide insight into the mechanism of viral rebound after treatment withdrawal even in long-term virally suppressed patients^15,22^.

Similar to Lai et al., we found very low levels of intrahepatic 3.5 kb RNA and 3.5 Kb RNA/cccDNA in this cohort of long-term telbivudine-treated patients with respect to previously published cohorts of untreated patients^14,23–25^. Therefore, we were interested in investigating the epigenetic status of the residual intrahepatic cccDNA.

An adaptation of the cccDNA-ChIP technique^5^ to small biopsy samples (micro-ChIP)^26^ allowed the identification of an enrichment of histone PTMs associated with inactive transcription on cccDNA derived from telbivudine-treated patients. Our observation of decreased histone H3 acetylation is in accordance with previous data obtained in vivo in the context of reduced cccDNA transcriptional activity in untreated patients^5,8,9^. Ren et al.^9^ also found an increased enrichment for H3K9me3 in inactive carrier CHB patients having low 3.5 kb RNA levels, while Flecken et al*.*^10^ failed to find such a difference for trimethylation on both H3 lysine 9 and 27 across patients belonging to different CHB phases. In the same study from Flecken et al., three NUC-treated patients were also analysed, but could not be differentiated from non-treated ones on the basis of cccDNA-associated histone PTMs enrichment profile ^10^. Our data showed that in this telbivudine-treated patient population with low 3.5 kb RNA/cccDNA ratio, H3K9me3 and H3K27me3 are enriched on histones associated with cccDNA.

Whether these changes in cccDNA-associated histone PTMs profile could be due to a direct effect of telbivudine-treatment remains to be investigated. The primary mode of action of NUCs, *i.e.* inhibition of HBV polymerase reverse transcriptase activity, would exclude any direct effect on cccDNA epigenome. However, one might hypothesize that a prolonged suppression of pgRNA to rcDNA conversion might trigger a negative feedback regulation of cccDNA transcription. On the other hand, we cannot rule out the hypothesis that the inhibition of cccDNA activity could be due to an additional property of telbivudine. Indeed, telbivudine was shown to modify host histone PTMs at specific loci in HepG2.2.15 cells, by restoring H3K4me3 and H3K27me3 to levels comparable to those detected in HepG2 cells^27^. Moreover, it has been shown that HBx protein levels may be reduced after telbivudine treatment in vitro^28^. Since HBx is essential for full cccDNA transcription and HBx-deficient HBV mutant strains show enrichment in cccDNA-associated histone PTMs correlated with inactive transcription^29^, this could represent an additional antiviral mechanism of telbivudine. If this effect on cccDNA epigenome is shared with other NUCs, or with emerging new antivirals, remains to be investigated. In this respect, recent data obtained by Balagopal et al. using single-cell laser capture microdissection and ddPCR in liver biopsies from HIV/HBV co-infected patients under antiviral therapy (DAART or ART) confirmed the decline of cccDNA amount along with the duration of the antiviral treatment and also observed a sharp decrease in preC/pgRNA levels in samples derived from long term-treated patients^30^. They also showed an increased number of cccDNA-positive hepatocytes without a detectable preC/pgRNA, suggesting an inactivation of cccDNA transcriptional activity correlating with long-term antiviral therapy^30^. Overall, their results could be consistent with our observation of an epigenetic repression of cccDNA during long-term NUC therapy. Further single-molecule studies would be required to better clarify the relative contribution to the 3.5 Kb RNA level decrease of cccDNA template decline *vs* epigenetic repression.

Of course, it cannot be excluded that other liver microenvironment factors such as changes in the inflammatory status and in cytokine expression during therapy may also be involved in this phenomenon.

Altogether, using a highly sensitive cccDNA quantification method together with the analysis of the cccDNA-associated epigenome, our data showed the persistence of low levels and epigenetically-modified intrahepatic cccDNA in patients with long-term telbivudine-induced viral suppression. Whilst the use of liver biopsies does not prevent a sampling bias due to potential focal differences in the infected tissue, our results underline the importance of implementing more sensitive techniques to quantify cccDNA in patients and to assess the epigenetic status and transcriptional activity of the viral minichromosome. This panel of cccDNA evaluation techniques should provide an added value for the new proof of concept clinical trials aiming at a functional cure of chronic hepatitis B.

## Patients and methods

### Patients

Fifty-six patients with chronic hepatitis B (CHB) (46 males and 10 females) previously enrolled in the clinical studies NV-02B-007 (NCT00057265) or NV-02B-015 (NCT00131742) and who entered the CLDT600ACN04E1 (NCT00877149) study (Novartis Pharma AG) without discontinuation of telbivudine treatment were included in this study (Supplementary Fig. 1)^31–33^. The studies were approved by each local independent ethics committee and were in accordance with the ethical standards of the Helsinki Declaration of 1975, as revised in 2000 and 2008. Written informed consent was obtained from each patient and/or their legal guardians.

Histological, clinical and virological data reported in this study were collected in studies NV-02B-007 or NV-02B-015 at baseline and after five years of antiviral treatment (Supplementary Fig. 1). Part of the liver specimens collected at CLDT600ACN04E1 study entry was snap frozen at − 80 °C for molecular biology analysis. These legacy clinical studies have been published with ethics committee approvals previously disclosed and publicly available^31–34^.

For the analysis of cccDNA histone post-translational modifications, 7 untreated CHB patients from a historical cohort collected at Hospices Civils de Lyon (Lyon’s University, France) were used as comparative samples. These patients underwent liver biopsy as part of their clinical follow-up, a fragment was preserved for research purposes and stored at − 80 °C. The protocol was approved by the competent Institutional Ethics Committee (CPP Sud est IV 11/040, authorization number DC-2008-235). Written informed consent was obtained from all patients and/or their legal guardians to underwent liver biopsy. No patients were co-infected with HIV, hepatitis C virus or hepatitis delta virus.

This submitted article is based on data from the previously submitted studies and does not involve any new studies or new human subjects. All methods were carried out in accordance with relevant guidelines/regulations. No animal experimentation and no use of primary human cells was employed in this study.

### Viral load and serological assessment

Serum HBV DNA, alanine aminotransferase (ALT) levels, HBe antigen (HBeAg), HBs antigen (HBsAg) and HBe and HBs serology were analyzed in the frame of the clinical trials by the study sponsor. Serum HBV DNA levels were quantified with the Roche COBAS Amplicor PCR assay (Roche Molecular Systems, Branchburg, NJ, USA), with an estimated lower limit of detection and quantification of 300 HBV genome copies/mL (around 60 IU/mL). Other HBV serologic markers (HBsAg/anti-HBs, HBeAg/anti-HBe) were assessed using standard commercially available assays^31,32^.

### Intrahepatic total HBV DNA, cccDNA and preC/pgRNA quantification using qPCR method

DNA was extracted from snap-frozen liver biopsies using the Master Pure DNA Purification Kit (Lucigen, Middleton, WI, USA) according to the manufacturer’s instructions. High Pure RNA Paraffin Kit (Roche Life Science, Indianapolis, IN, USA) was used for RNA extraction. Quantity and integrity of the extracted DNA and RNA were assessed by NanoDrop Spectrophotometer (Nanodrop Technologies, Wilmington, DE, USA). Patients’ samples were run in duplicate and in two independent experiments. Quantitative PCR were performed using specific primers and fluorescence hybridization probes previously described^17^ and a Light Cycler 480 Real Time PCR System (Roche Diagnostics, Manheim, Germany)^24,25^. Before cccDNA quantification, total DNA was treated with Plasmid-safe DNAse (LUCIGEN, Middleton, WI, USA), to limit relaxed circular DNA (rcDNA) contamination. Serial dilutions of an HBV monomer plasmid (pHBV-*Eco*R1) were used as standard for quantification. Beta-globin quantification was performed in parallel to assess the amount of HBV DNA copies/cell ^17^. Intrahepatic 3.5kB RNA (preC/pgRNA) quantification, consisting of both preCore and pgRNA transcripts, was performed with specific primers and TaqMan fluorescence hybridization probes previously described by Volz et al. ^23^. Results were normalized over the housekeeping gene GUSb (Hs00939627_m1, Thermofischer Scientific, Waltham, MA, USA). All the primers and probes used are listed in Supplementary Table 5 and visualized on HBV genome in Supplementary Fig. 5.

### cccDNA detection using rolling circle amplification

Rolling Circle Amplification (RCA) was shown to be highly specific for complete circular forms of HBV genome and particularly adapted for the amplification of cccDNA in liver tissue with low levels of viral replication ^35^. We assessed the presence of intrahepatic cccDNA in qPCR-negative samples using this one-step PCR method without additional steps of completion and ligation, as previously described using primers RCA1-8 listed in Supplementary Table 5 and Supplementary Fig. 6 (Fig. 2a) ^35,36^. The amplification of circular HBV DNA was performed using Phi29 polymerase (New England Biolabs, Ipswich, MA, USA) for 21 h at 30 °C on total DNA extracted from frozen liver samples. RCA products were analysed according to two different methods to assess the presence of intrahepatic cccDNA in the samples. The first one, Southern blot after digestion of RCA products allowed us to control for RCA specificity but was not sufficiently sensitive for samples with small amount of intrahepatic cccDNA. The second one, full length-HBV genomic PCR on RCA products allowed us to detect the presence of cccDNA even at very low levels (Fig. 2) ^35^. For Southern blot processing, RCA products were digested for 4 h at 37 °C with the restriction enzyme SpeI (NEW ENGLAND BIOLABS, Ipswich, MA, USA): final volume 10 µL, RCA products 4 µL, SpeI 3U and Cutsmart Buffer 1.1 µL (New England Biolabs, Ipswich, MA, USA). Digestion products were run on 1% agarose gel and then transferred to perform hybridization with HBV-specific cold probes (Fig. 2b) ^37^.

Full-length HBV genomic PCR amplification used primers P1 and P2 ^38^ (Supplementary Table 5 and Supplementary Fig. 6) to enhance cccDNA detection sensitivity in samples with low HBV DNA levels (Fig. 2c). The final PCR reaction included 2µL of RCA products, 0.25 µL of each sense and anti-sense primers previously described (100 µM) and 25 µL of PrimeSTAR HS (Premix) (Takara Bio Inc. Shiga, Japan) in a 50 µL final volume. The amplification protocol was adapted from Günther et al.^38^ and was performed for 40 cycles as follow: denaturation at 94 °C for 40 s; 1 min annealing at 55 °C for 10 cycles, then at 60 °C for 10 cycles and finally at 62 °C for the last 20 cycles; elongation at 68 °C for 3 min with an increment of 2 min after each 10 cycles. PCR products were run on 1% agarose gel.

### Intrahepatic total HBV DNA and cccDNA quantification by droplet digital PCR

Intrahepatic total HBV DNA and cccDNA were quantified by the QX100 Droplet Digital PCR System (Bio-Rad, Hercules, CA, USA) according to the manufacturer’s instructions. Before cccDNA quantification, total DNA was treated with Plasmid-safe DNAse (Lucigen, Middleton, WI, USA), to limit rcDNA contamination. Briefly, the 25 μL ddPCR reaction was comprised of 2× ddPCR Supermix for probes (Bio-Rad, Hercules, CA, USA), 900 nM HBV cccDNA forward and reverse primers, 250 nM HBV probe, and 5 μL of DNA sample. The plates were loaded with required consumables into the Automated Droplet Generator to partition the sample around 20,000 droplets in 20 µL. PCR amplification was performed in a C1000 Touch thermal cycler (Bio-Rad, Hercules, CA, USA) with the following amplification program: 10 min at 95 °C, 40 cycles of denaturation for 30 s at 94 °C and annealing for 60 s at 60 °C (ramping rate set to 2 °C/s), final incubation step for 10 min at 98 °C. After reading in a QX100 Droplet Reader (Bio-Rad, Hercules, CA, USA), the data were analyzed using the QuantaSoft analysis software (Bio-Rad, Hercules, CA, USA), which automatically calculated absolute sample concentration after applying Poisson’s distribution. Fluorescence amplitude threshold to distinguish positive from negative droplets was based on amplification of negative controls (water and non-HBV infected samples, Fig. 3). Concomitant quantification of HBV DNA and beta-globin in the same sample allowed the estimation of HBV DNA or cccDNA copies/cell. All primers and probes used are listed in Supplementary Table 5 and Supplementary Fig. 5. The pBR322-HBV construct was used to assess linearity of ddPCR quantification and to compare it to qPCR. Limit of detection for cccDNA quantification in ddPCR was 4.8 copies/10^6^ cell (3.3–7, 95% confidence interval), calculated by Probit analysis on a sample composed of serial dilution of a minicircle HBV into genomic DNA.

Specificity of cccDNA assay for episomal HBV DNA vs integrated sequences was analyzed in Hep3B cells, which harbor nearly 1 HBV integration/cell, but no cccDNA (Supplementary Methods and Supplementary Fig. 3a,b). cccDNA assay was specific for cccHBV DNA *vs* rcDNA if the rcDNA:cccDNA ratio was < 1000 without the need of prior PSD digestion (Supplementary Fig. 3c). In HBV-infected HepG2-NTCP cells, NUC treatment by lamivudine (3TC) lowers the rcDNA:cccDNA to less than 100 and addition of PSD digestion ensures a rcDNA:cccDNA around 1, thus highly incrementing the specificity of our assay for cccDNA (Supplementary Methods and Supplementary Fig. 3d). Thus, these data supported the use of this ddPCR cccDNA protocol for specific cccDNA quantification over other HBV genomic forms in the long-term NUC-treated patients analyzed in this study.

### Chromatin immunoprecipitation from frozen liver biopsies

ChIPs from liver biopsies were performed as described by Testoni et al.^26^. Briefly, frozen biopsies were removed from the freezer and immediately added with PBS/1% formaldehyde and incubated 10 min at room temperature. Crosslinking reaction was quenched with 0.125 M glycine. After centrifugation, the cross-linked biopsy was resuspended in lysis buffer (50 mM Tris–HCl pH 8, 10 mM EDTA, 1% SDS, protease inhibitors) and homogenized with the help of a pestle. Supernatant was then sonicated in a Bioruptor (DIAGENODE, Liege, Belgium) for 2 × 30 s cycles, diluted 1:10 in Ripa Buffer (10 mM Tris–HCl pH 7.5, 140 mM NaCl, 1 mM EDTA, 0.5 mM EGTA, 1% Triton X-100, 0.1% SDS, 0.1% Na-deoxycolate, protease inhibitors) and incubated with Dynabeads Protein G and 3 μg of each antibody (ChIP-grade anti-H3K27Ac, H3K56Ac, H3K9me3, H3K27me3, DIAGENODE, Liege, Belgium) overnight at 4 °C rotating. After immunoprecipitation, washes and reverse crosslinking, the samples were extracted twice with phenol/chloroform, once with chloroform and ethanol precipitated in the presence of 30 μg of glycogen (SIGMA ALDRICH-MERCK, Darmstadt, Germany). Quantitative PCR was performed using cccDNA-specific primers and probes. Glyceraldehyde-3-phosphate Dehydrogenase (GAPDH) promoter region amplification served as control for specific enrichment of antibodies (see Supplementary Table 5 and Supplementary Fig. 4a).

### Statistical analyses

Statistical analysis was performed using Prism 7 software (GRAPHPAD SOFTWARE, San Diego, CA, USA). Mann Whitney U Test and Kruskal Wallis test were used to compare numerical data and Chi-square test was used to compare frequencies between groups, with Fisher’s exact test correction if appropriate. Probit analysis was performed using SPSS 18.0 (SPSS Inc, Chicago, IL).

## Supplementary information

Supplementary Information

## Abbreviations

ALT: Alanine aminotransferase
cccDNA: Covalently closed circular DNA
CHB: Chronic hepatitis B
ChIP: Chromatin immunoprecipitation
ddPCR: Droplet digital PCR
HBeAg: Hepatitis B e Antigen
HBsAg: Hepatitis B s Antigen
HBV: Hepatitis B virus
HCC: Hepatocellular carcinoma
HIV: Human immunodeficiency virus
NUCs: Nucleos(t)ides analogues
pgRNA: Pre-genomic RNA
preC/pgRNA: PreCore and pre-genomic RNA
PTMs: Post-transcriptional modifications
qPCR: Quantitative PCR (polymerase chain reaction)
RCA: Rolling circle amplification
rcDNA: Relaxed circular DNA
ULN: Upper limit of normal
