# Supplementary material for: Quantification and epigenetic evaluation of the residual pool of hepatitis B covalently closed circular DNA in long-term nucleoside analogue-treated patients
Source: Sci Rep. 2020 Dec 3;10:21097. doi: 10.1038/s41598-020-78001-1 (PMC7712874; doi:10.1038/s41598-020-78001-1)
Supplement: Supplementary file 1 — Supplementary Information [file 41598_2020_78001_MOESM1_ESM.pdf]

Quantification and epigenetic evaluation of the residual pool of hepatitis B covalently closed circular DNA in long-term nucleoside analogue-treated patients

Fanny Lebossé, Aurore Inchauspé, Maëlle Locatelli, Clothilde Miaglia, Audrey Diederichs, Judith Fresquet, Fleur Chapus, Kamal Hamed, Barbara Testoni and Fabien Zoulim

Supplementary information content

Supplementary Methods

Supplementary Table 1

Supplementary Table 2

Supplementary Table 3

Supplementary Table 4

Supplementary Table 5

Supplementary Figure 1

Supplementary Figure 2

Supplementary Figure 3

Supplementary Figure 4

Supplementary Figure 5

Supplementary Figure 6

Supplementary References

Original blots referring to Main Figure 2

Supplementary Methods

Production of HBV viral inoculum

HBV inoculum was prepared from filtered HepAD38 cells[1] supernatants by polyethylene-glycol-MW-8000 (PEG8000, SIGMA ALDRICH-MERCK, Darmstadt, Germany) precipitation (8% final) as previously described [2]. Viral stock with a titer reaching at least $1x10^{10}$ viral genome equivalents (vge)/mL was tested endotoxin free and used for infection.

Extraction of virion-associated rcDNA from HepAD38 supernatant

HBV inoculum was PEG-precipitated and DNA extracted with High Pure Viral Nucleic Acid kit (ROCHE LIFE SCIENCE, Indianapolis, IN, USA) and HBV DNA quantified using tHBV DNA assay listed in Supplementary Table 5.

Cell culture, HBV infection and treatments

Hep3B cells were maintained DMEM medium supplemented with 1% penicillin/streptomycin (THERMOFISCHER SCIENTIFIC, Waltham, MA, USA), 1% glutamine (THERMOFISCHER SCIENTIFIC, Waltham, MA, USA). HepG2-NTCP cells were seeded at 10^5 cells/cm2 in DMEM medium supplemented with 1% penicillin/streptomycin (THERMOFISCHER SCIENTIFIC, Waltham, MA, USA), 1% sodium pyruvate (THERMOFISCHER SCIENTIFIC, Waltham, MA, USA), 5% Fetal Calf Serum (FCS; Fetalclone IITM, PERBIO, THERMOFISCHER SCIENTIFIC, Waltham, MA, USA). The day after, medium was renewed and complemented with 2.5% DMSO (SIGMA ALDRICH-MERCK, Darmstadt, Germany). After 72h, cells were infected at a multiplicity of infection of 250 in the presence of 4% PEG8000 for up to 16h and then extensively washed and cultured in complete DMEM medium containing 2.5% DMSO until harvesting. 3 days post-infection, lamivudine (3TC) was added to the cell culture medium at a final concentration of 10µM and treatment renewed every day until cells harvesting.

Minicircle HBV production

The plasmid pMC-HBV containing the HBV sequence of the strain ayw has been obtained as described in [3]. Plasmid DNA was extracted using the Nucleobond Xtra Maxi endotoxin free kit according to the manufacturer's instruction (MACHEREY-NAGEL, Bethlehem, PA, USA) and digested by the NdeI (NEW ENGLAND BIOLABS, Ipswich, MA, USA) restriction enzyme for 2 h at 37 °C and by a Plasmid-Safe DNase (LUCIGEN, Middleton, WI, USA) overnight at

37 °C. After purification, the resulting plasmid has been observed on agarose gel to check for the elimination of the parental plasmid. Devoid of any bacterial backbone, the resulting minicircle served as a surrogate for authentic cccDNA to test the specificity of the ddPCR amplification assay (Supplementary Figure 3c).

Supplementary Table 1

Serum and intrahepatic markers according to HBeAg loss or seroconversion at the time of liver biopsy[a]

	**HBeAg loss at the time of liver biopsy [b] n=28**	**No HBeAg loss at the time of liver biopsy [b] n=9**	**p[c]**	**HBeAg seroconversion at the time of liver biopsy [b] n=15**	**No HBeAg seroconversion at the time of liver biopsy [b] n=22**	**p[c]**
**Baseline Age** (years)	27 (21 – 36)	28 (25 -33)	ns	25 (18 – 32)	29 (25 -39)	ns
**Baseline Serum HBV DNA** (IU/mL)	3.5×10^{8} (4.5×10^{7} – 1.5×10^{9})	2.3×10^{7} (6.5×10^{5} – 2.5×10^{8})	ns	1.8×10^{8} (4.3×10^{7} – 1×10^{9})	2.5×10^{8} (4.9×10^{6} – 1.7×10^{9})	ns
**Baseline ALT level** (IU)	1.7×10^{2} (1.3×10^{2} - 2.5×10^{2})	1×10^{2} (7.9×10^{1} – 3.3×10^{2})	ns	1.7×10^{2} (1.4×10^{2} - 2.4×10^{2})	1.4×10^{2} (9×10^{1} – 3.1×10^{2})	ns
**Duration of telbivudine treatment** (weeks)	260 (157 – 261)	165 (157 – 260)	ns	172 (157 – 261)	234 (157 – 261)	ns
**Liver biopsy intrahepatic total HBV DNA** (copies/cell)	1.5×10^{-1} (8.6×10^{-2} – 2.5×10^{-1})	2.6×10^{-1} (1×10^{-1} – 3.1×10^{-1})	ns	2.2×10^{-1} (6.2×10^{-2} – 2.9×10^{-1})	1.5×10^{-1} (9.9×10^{-2} – 2.3×10^{-1})	ns
**Liver biopsy intrahepatic cccDNA [d, e]** (Detectable / undetectable)	12 / 16	3 / 6	ns	6 / 9	9 / 13	ns
**Liver biopsy intrahepatic 3.5Kb RNA [e]** (relative quantity)	1.5×10^{-1} (6×10^{-2} – 4.8×10^{-1})	5.7×10^{-1} (1.3×10^{-1} – 1.1×10^{0})	ns	1.1×10^{-1} (3.4×10^{-2} – 5.1×10^{-1})	3.1×10^{-1} (8.7×10^{-2} – 6.8×10^{-1})	ns

[a] Data for baseline HBeAg(+) patients (n=37); [b] data are expressed as median (1st percentile-3rd percentile); [c] Mann Whitney U test between groups; [d] χ^2 test between groups; [e] Results for viral DNA and RNA were obtained using the qPCR quantification method.

Supplementary Table 2

ddPCR quantification of intrahepatic cccDNA

HBe Ag loss at liver biopsy [a]		p value [b]
**HBe Ag loss (n=23)**	**No HBe Ag loss (n=8)**	
7.3×10^{-3} ($2.6 \times 10^{-3} – 1.9 \times 10^{-2}$)	9.3×10^{-3} ($3.2 \times 10^{-3} – 2.5 \times 10^{-2}$)	ns
**HBe seroconversion at liver biopsy [a]**		**p value [b]**
**HBe seroconversion (n=13)**	**No HBe seroconversion (n=18)**	
8.3×10^{-3} ($2.3 \times 10^{-3} – 2.3 \times 10^{-2}$)	4.5×10^{-3} ($2.9 \times 10^{-3} – 1.8 \times 10^{-2}$)	ns

[a] data available for 31 patients; [b] Mann Whitney U test between groups

Supplementary Table 3

Demographical and virological characteristics of Telbivudine-treated patients analyzed for cccDNA epigenetic status compared to Telbivudine-treated patients not included in ChIP analysis

	**Telbivudine patients analyzed by ChIP (cccDNA detectable by qPCR) (n=10)**	**Telbivudine patients not included in ChIP analysis (n=34, cccDNA undetectable by qPCR but quantifiable by ddPCR)**	**p-value**
**Baseline**			
**Age** (years) [a,b]	29<br>(26 – 35)	33<br>(27 – 42)	ns
**serum HBV DNA** (logIU/ml) [a,b]	1.6×10^{8}<br>($1.1 \times 10^{6} – 1.9 \times 10^{9}$)	4.8×10^{7}<br>($4 \times 10^{5} – 4.8 \times 10^{8}$)	ns
**ALT levels** (IU/L) [a,b]	166.5<br>(105.3 – 188)	151.5<br>(90.2 – 319.3)	ns
**Duration of treatment** (weeks) [a,b]	168.5<br>(157 – 260)	260<br>(158 – 261)	ns
**Genotype**[c]<br>**B**<br>**C**	 3<br>7	 6<br>28	ns
**At liver biopsy**			
**Serum HBV DNA**[c]<br>(detectable/undetectable/missing)	2 / 7 / 1	0 / 33 / 1	-
**HBeAg status** (P/N)[c]	4 / 6	5 / 29	ns
**ALT levels** (IU/L) [a,b]	20.5<br>(14.3 – 30.9)	28<br>(21 – 48.5)	0.03
**Intrahepatic total HBV DNA** [a,b]<br>(copies/cell)	2.6×10^{-1}<br>($1.1 \times 10^{-2} – 3.4 \times 10^{-1}$)	9.8×10^{-2}<br>($3.8 \times 10^{-2} – 2.3 \times 10^{-1}$)	ns
**Intrahepatic cccDNA** [a,b,d]<br>(copies/cell)	2.4×10^{-1}<br>($1.1 \times 10^{-1} – 3.4 \times 10^{-1}$)	9×10^{-3}<br>($3.7 \times 10^{-3} – 1.7 \times 10^{-2}$)	<0.0001
**Intrahepatic 3.5Kb RNA** [a,b]<br>(relative quantity)	3.7×10^{-1}<br>($8.4 \times 10^{-2} – 9.8 \times 10^{-1}$)	2×10^{-1}<br>($5.4 \times 10^{-2} – 6 \times 10^{-1}$)	ns
**Intrahepatic 3.5Kb RNA/cccDNA** [a,b,d]<br>(relative quantity)	6.03<br>(2.3 – 37.5)	24.1<br>(3.9 – 130.9)	ns

[a] Mann Whitney U test between each group; [b] data are expressed as median (1st percentile-3rd percentile); [c] $\chi 2$ test between each group; [d] cccDNA quantification was performed by ddPCR assay

Supplementary Table 4

Demographical and virological characteristics of patients analyzed for cccDNA epigenetic status

	**Telbivudine-treated patients (n=10)**	**Untreated CHB Comparative group (n=7)**	**p**
**Age** [a, b] (years)	29 (26 - 35)	22 (19 - 34)	ns
**Genotype** [c] **(B / C / D)**	3 / 7 / 0	1 / 5 / 1	ns
**HBeAg** [c, d] (+/-)	4 / 6	5 / 2	ns
**Intrahepatic total HBV DNA** [a, b, e] (copies/cell)	2.6×10^{-1} ($1.1 \times 10^{-2} – 3.4 \times 10^{-1}$)	7.1×10^{1} ($3.8 \times 10^{1} – 2.3 \times 10^{2}$)	0.0001
**Intrahepatic cccDNA** [a, b, e] (copies/cell)	2.4×10^{-1} ($1.1 \times 10^{-1} – 3.4 \times 10^{-1}$)	2.7×10^{0} ($9.5 \times 10^{-1} – 3.2 \times 10^{0}$)	0.0001
**3.5Kb RNA** [a, b, e] (relative quantity)	3.7×10^{-1} ($8.4 \times 10^{-2} – 9.8 \times 10^{-1}$)	1.6×10^{2} ($1.5 \times 10^{2} – 1.7 \times 10^{3}$)	0.0001
**3.5Kb RNA/cccDNA** [a,b] (relative quantity)	6.03 (2.3 – 37.5)	160 (56.3 – 237)	0.0001

[a] Mann Whitney U test between each group; [b] data are expressed as median (1st percentile-3rd percentile); [c] χ^2 test between each group; [d] HBeAg status at the time of liver biopsy; [e] intrahepatic viral nucleic acid markers quantification was performed by qPCR assay

Supplementary Table 5

List of primers and probes used in the study

Target and technique	Oligos Name	Sequence 5'-3'	Position on HBV[a]
**tHBV DNA qPCR (FRET chemistry)[b]**	tHBV FRET for	CTCGTGGTGGACTTCTCTC	255-273
	tHBV FRET rev	CAGCAGGATGAAGAGGAA	403-420
	tHBV FRET FL	CACTCACCAACCTCCTGTCCTCCAA(FL)	334-358
	tHBV FRET LC	(R_{640})TGTCCTGGTTATCGCTGGATGTGTCT	361-386
**cccDNA qPCR (FRET chemistry)[b]**	cccDNA FRET for	CTCCCCGTCTGTGCCTTCT	1547-1565
	cccDNA FRET rev	GCCCCAAAGCCACCCAAG	1885-1902
	cccDNA FRET FL	GTTCACGGTGGTCTCCATGCAACGT(FL)	1603-1627
	cccDNA FRET LC	(R_{640})AGGTGAAGCGAAGTGCACACGGACC	1572- 1596
**cccDNA ddPCR and ChIP (TaqMan chemistry)[c]**	cccDNA TaqMan for	CCGTGTGCACTTCGCTTCA	1577-1595
	cccDNA TaqMan rev	GCACAGCTTGGAGGCTTGA	1866-1884
	cccDNA TaqMan probe	(6FAM)CATGGAGACCACCGTGAACGCCC(BBQ)	1609-1631
**3.5Kb RNA qPCR (TaqMan chemistry)[c]**	3.5Kb RNA TaqMan for	GGAGTGTGGATTCGCACTCCT	2269-2289
	3.5Kb RNA TaqMan rev	AGATTGAGATCTTCTGCGAC	2417-2436
	3.5Kb RNA TaqMan probe	(6FAM)AGGCAGGTCCCCTAGAAGAAGAACTCC(BBQ)	2358-2384
**β-globin qPCR (FRET chemistry)[b]**	β-globin FRET for	ACACAACTGTGTTCACTAGC	
	β-globin FRET rev	CAACTTCATCCACGTTCACC	
	β-globin FRET FL	CAAACAGACACCATGGTGCACCTGACTCCTGAGGA(FL)	
	β-globin FRET LC	(R_{640})AAGTCTGCCGTTACTGCCCTGTGGGGCAA	
**β-globin ddPCR (TaqMan chemistry)[c]**	β-globin TaqMan	Life Technologies commercial assay Ref. Hs00758889_s1	
**GUSb qPCR (TaqMan chemistry)[c]**	GUSb TaqMan	Life Technologies commercial assay Ref. Hs00939627_m1	
**HBV RCA**	RCA1	AATCCTCACAATA*C*C	226-240
**HBV RCA**	RCA2	GATGGGATGGGAA*T*A	615-601
**HBV RCA**	RCA3	CCTATGGGAGTGG*G*C	637-651
**HBV RCA**	RCA4	GCAACGGGGTAAA*G*G	1154-1140
**HBV RCA**	RCA5	ATGCAACTTTTTC*A*C	1814-1828
**HBV RCA**	RCA6	TCCAAATTCTTTA*T*A	1930-1916

**HBV RCA**	RCA7	TAGAAGAAGAACT*C*C	2368-2382
**HBV RCA**	RCA8	AGAATATGGTGAC*C*C	2828-2814
**Full genome RCA**[d]	P1 rev	TTTTTCACCTCTGCCTAATCATC	1823-1845
	P2 for	AAAAAGTTGCATGGTGCTGGTG	1806-1827
**GAPDH promoter for ChIP**	GAPDH_for	CTTCTCCCCATTCCGTCTTC	
	GAPDH_rev	CCCCAGCTACAGAAAGGTCA	

[a] Nucleotide position is referred to EcoRI site on HBV sequence ayw, U9551.1

[b] published in Werle-Lapostolle et al.[4]

[c] published in Allweiss et al.[5]

[d] According to Günther et al.[6]

* phosphotioate modification

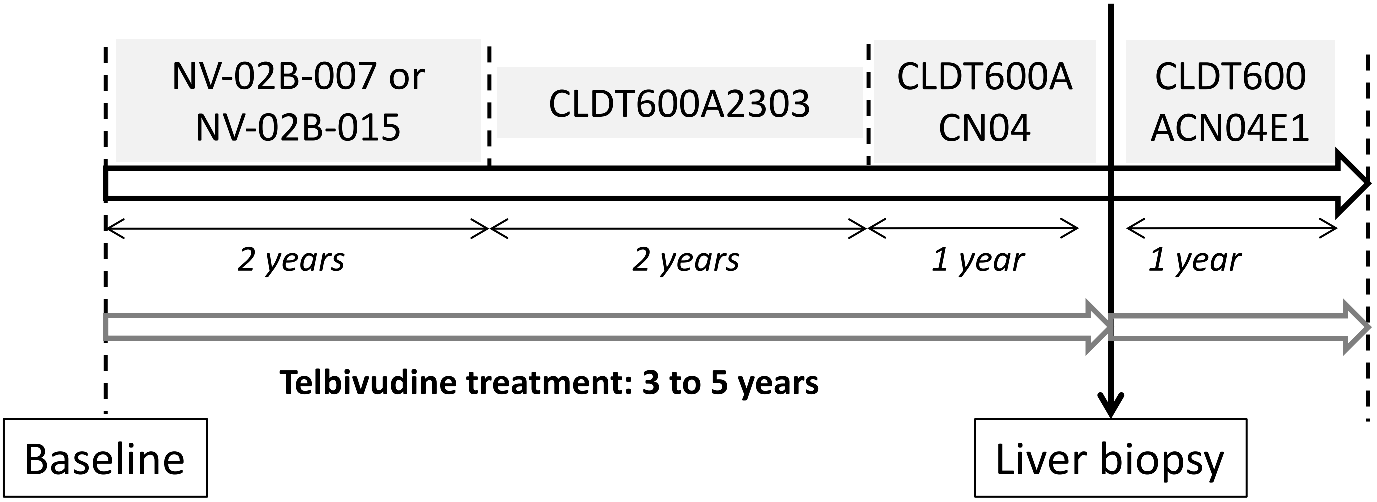

Supplementary Figure 1: Flow-chart of patients' selection. The fifty-six patients analyzed in the manuscript were first enrolled in the NV-02B-007 (GLOBE) or NV-02B-015 phase 3 clinical trials comparing telbivudine (600mg/day) *vs* lamivudine (100mg/day) treatment in patients with chronic hepatitis B. Then, they entered the GLOBE study extension CLDT600A2303 and the CLDT600ACN04E/E1 studies to assess the impact of longer telbivudine treatment on virological response and liver histological improvement. A liver biopsy was performed after 3 to 5 years of antiviral treatment, one week after inclusion in CLDT600ACN04E1 study (extension of telbivudine therapy) for histological and molecular virology analyses. These legacy clinical studies have been published with ethics committee approvals previously disclosed and publicly available[7-10]. This submitted article is based on data from the previously submitted studies and does not involve any new studies or new human subjects.

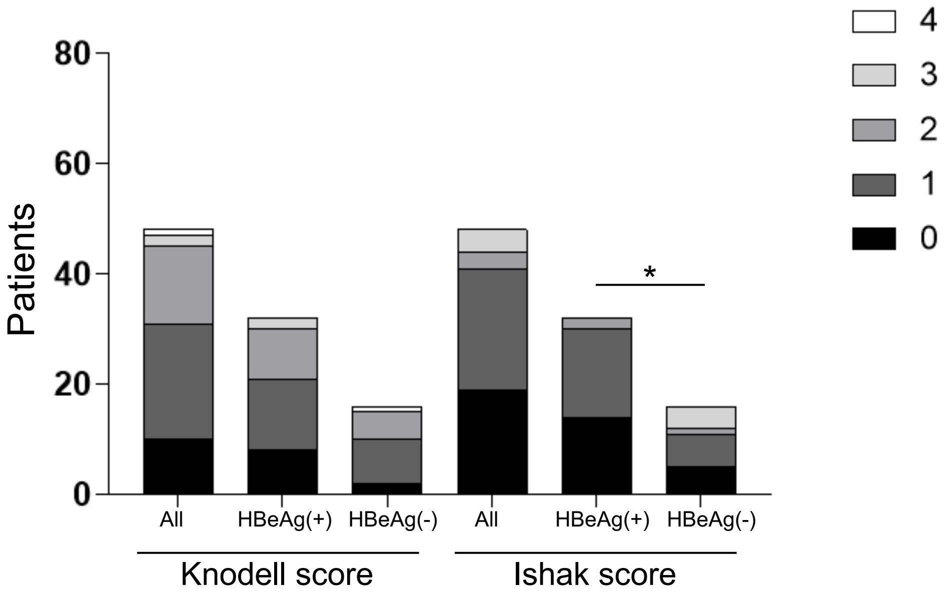

<u>Supplementary Figure 2</u>: Patients' distribution according to liver histological scores at baseline. Knodell activity and Ishak fibrosis scores were assessed at the inclusion in NV-02B-007 or NV-02B-015 clinical trials (Baseline, Supplementary Figure 1). * p<0.05 (Mann Whitney U test between HBeAg groups, alpha threshold = 0.05)

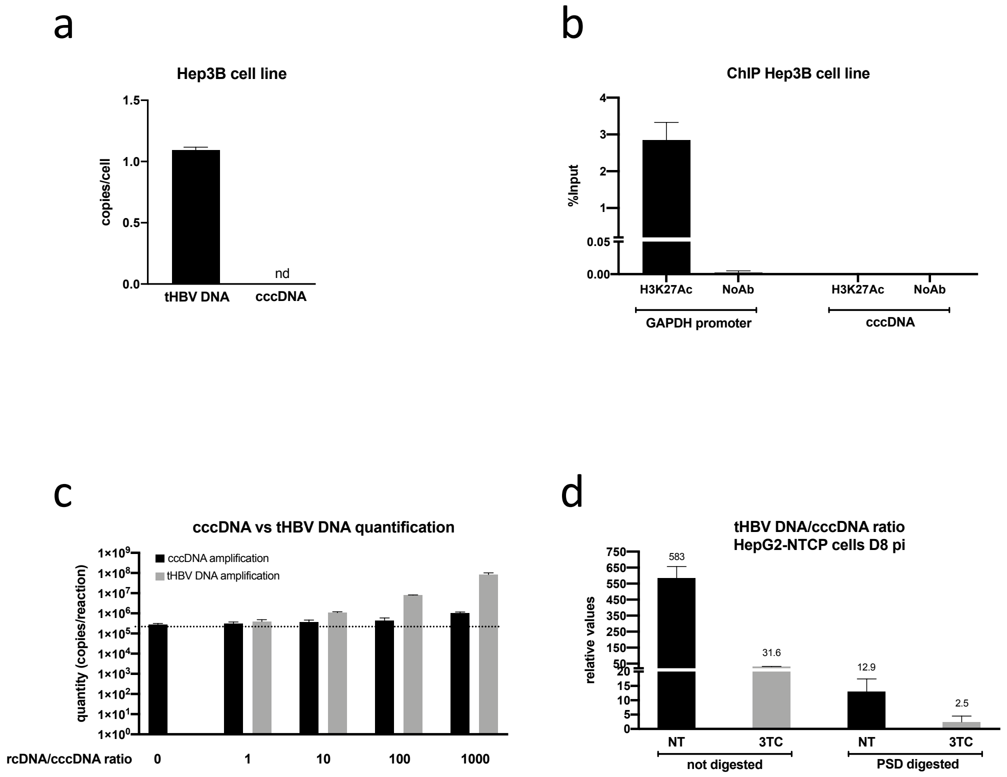

Supplementary Figure 3: cccDNA assay specifically amplifies episomal HBV DNA over integrated sequences and preferentially recognizes covalently-closed-circular DNA over rcDNA. **(a)** DNA was extracted from Hep3B cell line using the Master Pure DNA Purification Kit (LUCIGEN, Middleton, WI, USA) according to the manufacturer's instructions. **(b)** Chromatin Immunoprecipitation analysis was performed using a specific antibody against H3K27Ac. Signal enrichment is expressed as the percentage of input chromatin or cccDNA in Hep3B cell line. **(c)** 3 x 10^5 minicircle HBV molecules were mixed with increasing concentrations of rcDNA extracted from virions released in HepAD38 cell supernatant. The mix was quantified using cccDNA (black bars) or tHBV DNA (grey bars) assays. **(d)** HepG2-NTCP cells were infected with 250 viral genome equivalents (veg)/cell of a concentrated HBV inoculum derived from HepAD38 cells. Cells were treated (grey bars) or not (black bars) with 10µM 3TC everyday beginning from day 3 post-infection. Eight days post-infection, cells were harvested and intracellular DNA extracted with the Master Pure DNA Purification Kit (LUCIGEN, Middleton, WI, USA) according to the manufacturer's instructions. Before cccDNA quantification and where specified, DNA was digested with Plasmid Safe DNase (PSD) for 4h at 37°C followed by inactivation for 20 minutes at 70°C. NoAb: negative control; GAPDH: Glyceraldehyde-3-phosphate Dehydrogenase; PSD: Plasmid Safe DNase.

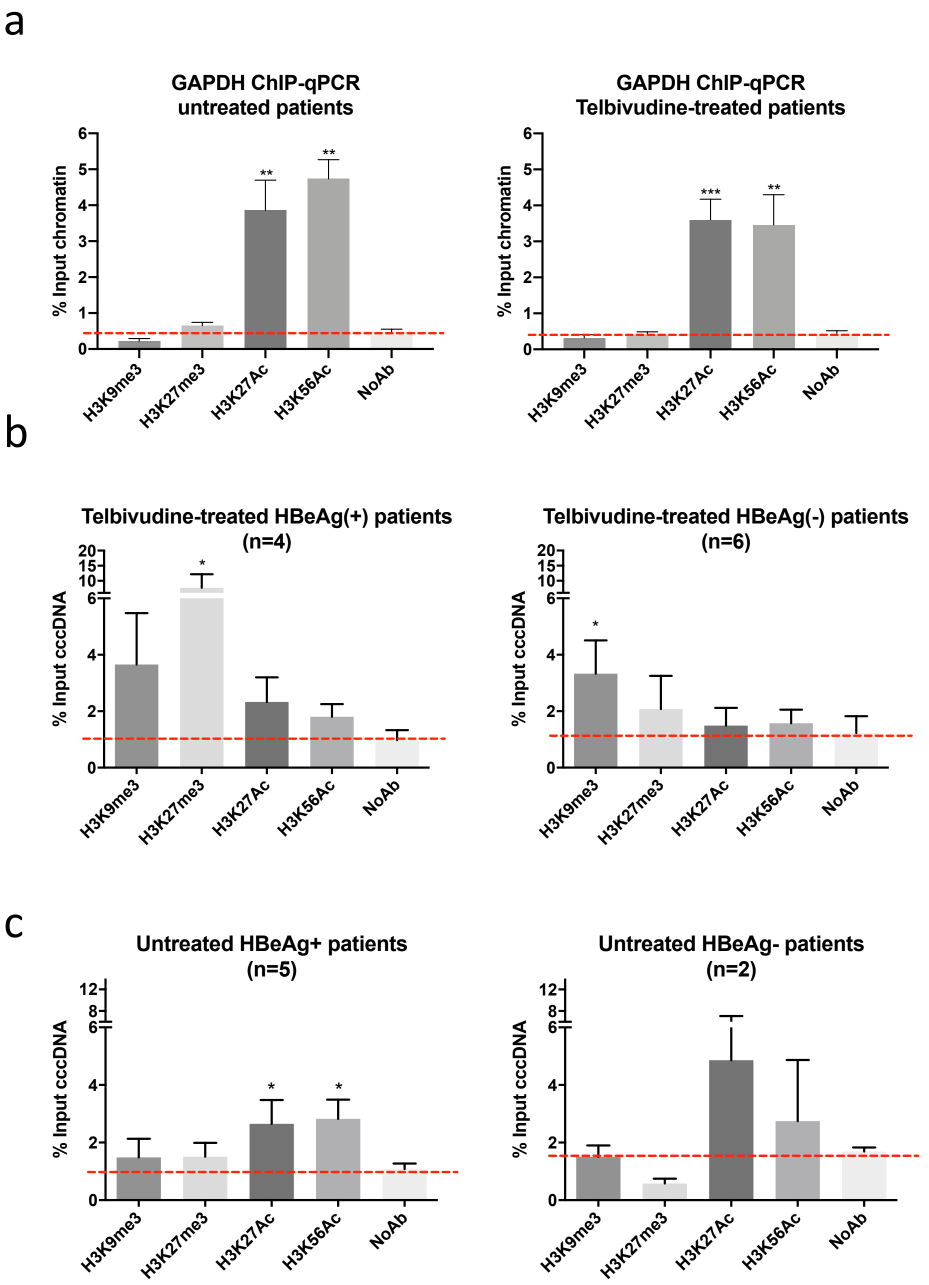

__**Supplementary Figure 4.**__ Chromatin Immunoprecipitation analysis was performed using specific antibodies against H3K9me3, H3K27me3, H3K27Ac and H3K56Ac. **(a)** Signal enrichment is expressed as the percentage of input chromatin in untreated chronic hepatitis B comparative group (n=7, left panel) and Telbivudine-treated patients (n=10, right panel). **(b-c)** Signal enrichment is expressed as the percentage of input cccDNA in Telbivudine-treated HBeAg(+) (n=4, left panel) and HBeAg(-) (n=6, right panel) patients **(b)** and in untreated HBeAg(+) (n=4, left panel) and HBeAg(-) (n=3, right panel) patients. Primers and probes for GAPDH promoter and cccDNA amplification are listed in Supplementary Table 5. Mann-Whitney U test was used to compare enrichment of specific antibodies *vs* the negative control (NoAb), alpha threshold = 0.05; * $p<0.05$. GAPDH: Glyceraldehyde-3-phosphate Dehydrogenase; cccDNA: covalently closed circular DNA, NoAb: negative control

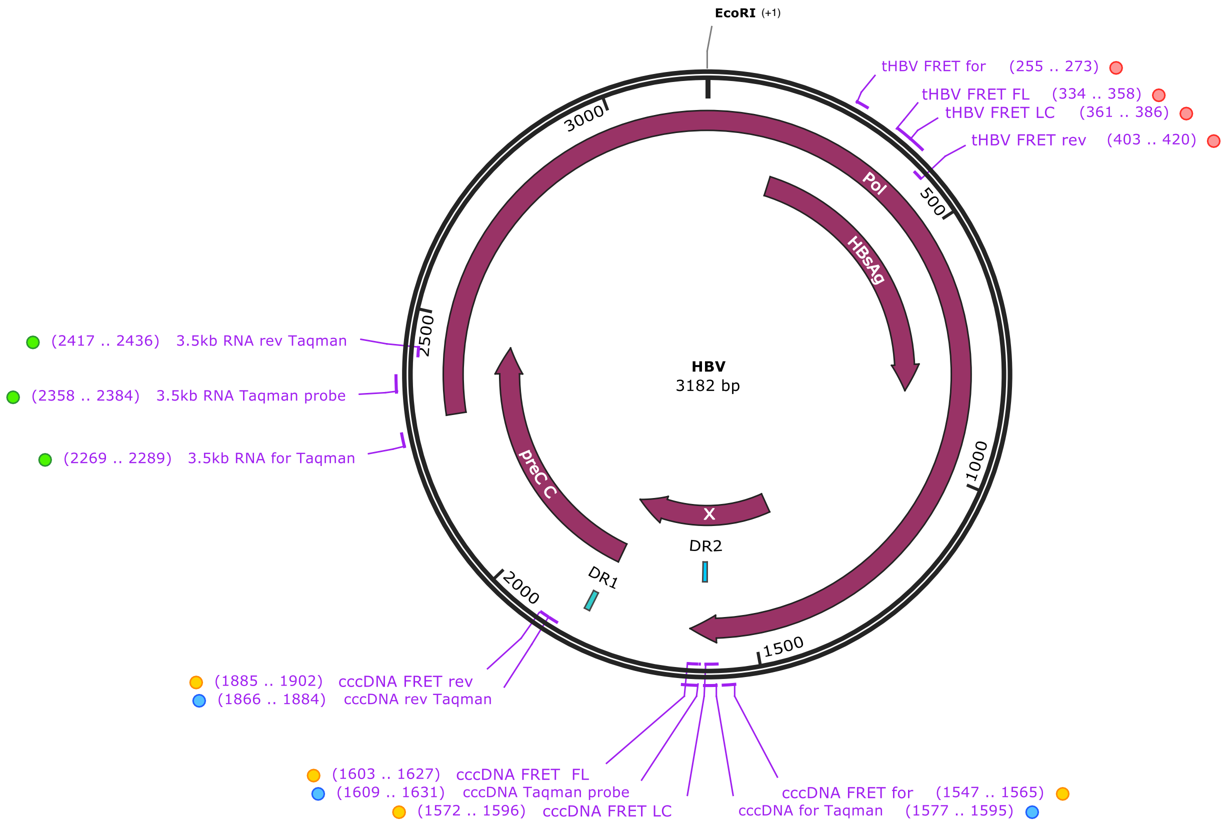

Supplementary Figure 5: Localization on HBV genome (strain ayw, U95551.1) of primers and probes used in the study. Green dots: primers and probes for 3.5Kb RNA quantification by qPCR; red dots: primers and probes for total HBV quantification by qPCR; yellow dots: primers and probes for cccDNA quantification by qPCR; blue dots: primers and probes for cccDNA quantification by ddPCR and by qPCR after chromatin immunoprecipitation (ChIP). Nucleotide positions are referred to EcoRI site.

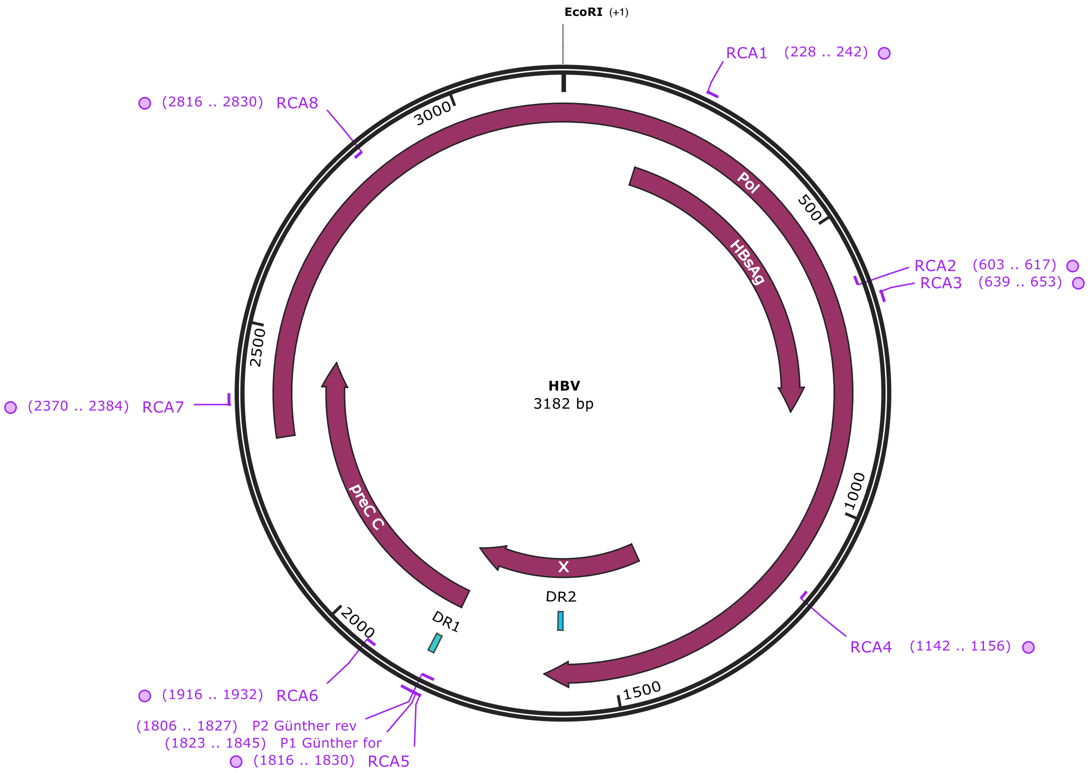

<u>Supplementary Figure 6</u>: Localization on HBV genome (strain ayw, U95551.1) of primers used for rolling circle amplification (RCA). Nucleotide positions are referred to EcoRI site.

Supplementary References

1. Ladner, SK *et al.* Inducible expression of human hepatitis B virus (HBV) in stably transfected hepatoblastoma cells: a novel system for screening potential inhibitors of HBV replication. *Antimicrob Agents Chemother.* **41(8)**, 1715-1720 (1997).

2. Luangsay, S. *et al.* Early inhibition of hepatocyte innate responses by hepatitis B virus. *J Hepatol.* **63(6)**, 1314-22 (2015).

3. Yan, Z. *et al.* HBVcircle: A novel tool to investigate hepatitis B virus covalently closed circular DNA. *J Hepatol.* **66(6)**, 1149-1157 (2017).

4. Werle-Lapostolle, B. *et al*. Persistence of cccDNA during the natural history of chronic hepatitis B and decline during adefovir dipivoxil therapy. *Gastroenterology* **126**, 1750–1758 (2004).

5. Allweiss, L. *et al*. Proliferation of primary human hepatocytes and prevention of hepatitis B virus reinfection efficiently deplete nuclear cccDNA in vivo. *Gut* **67**, 542–552 (2018).

6. Günther, S. *et al*. A novel method for efficient amplification of whole hepatitis B virus genomes permits rapid functional analysis and reveals deletion mutants in immunosuppressed patients. *J. Virol*. **69**, 5437–5444 (1995).

7. Hou, J. *et al*. Telbivudine versus lamivudine in Chinese patients with chronic hepatitis B: Results at 1 year of a randomized, double-blind trial. *Hepatol. Baltim. Md* **47**, 447–454 (2008).

8. Hou, J.-L. *et al*. Long-Term Telbivudine Treatment Results in Resolution of Liver Inflammation and Fibrosis in Patients with Chronic Hepatitis B. *Adv. Ther*. **32**, 727–741 (2015).

9. Lai, C.-L. *et al*. Telbivudine versus lamivudine in patients with chronic hepatitis B. *N. Engl. J. Med*. **357**, 2576–2588 (2007).

10. Zeuzem, S. *et al*. Baseline characteristics and early on-treatment response predict the outcomes of 2 years of telbivudine treatment of chronic hepatitis B. *J. Hepatol*. **51**, 11–20 (2009).

Southern blot on SpeI digested RCA products (Fig 2B)

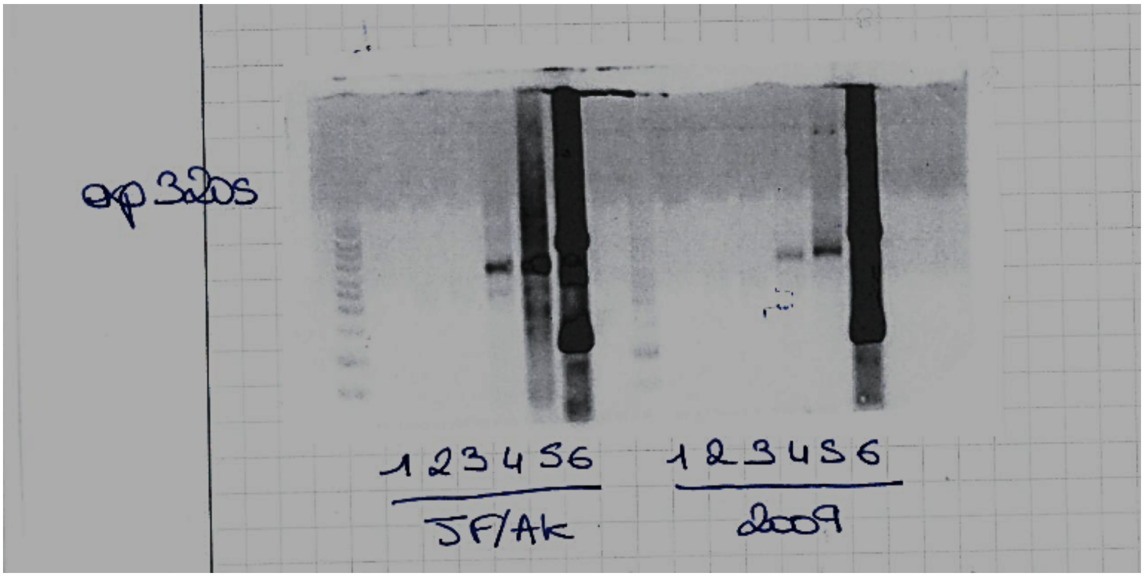

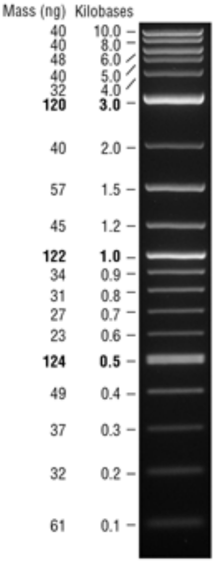

Left side of the gel has been used in Figure 2B. It represents an amelioration of RCA protocol used in the right side of the gel. Same samples were used for the two amplifications.

1 : Water
2 : sample with cccDNA concentration 0.01 copies/cell
3 : sample with cccDNA concentration 0.11 copies/cell
4 : sample with cccDNA concentration 1.2 copies/cell
5 : sample with cccDNA concentration 30 copies/cell
6 : positive control : HBV plasmid

Electrophoresis following P1P2 PCR (Fig 2C)

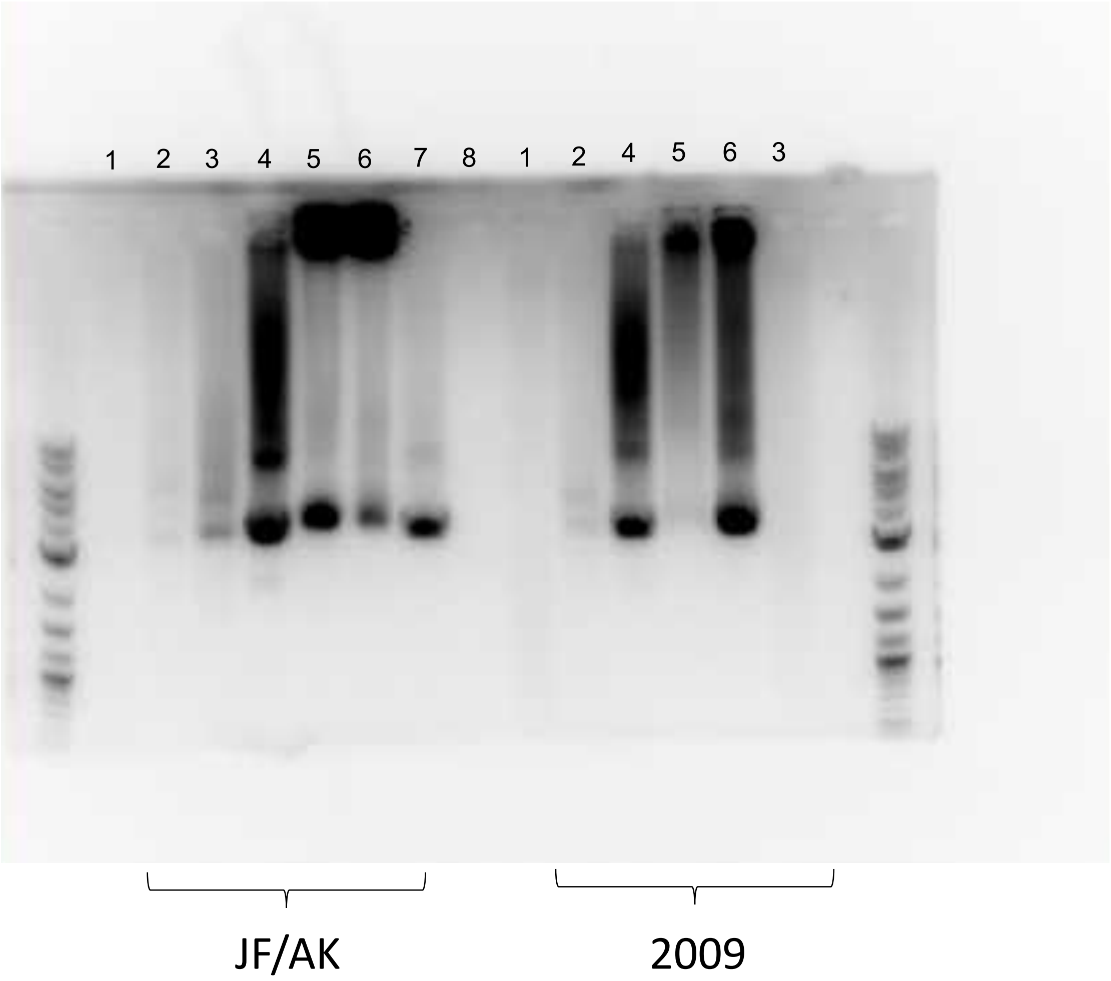

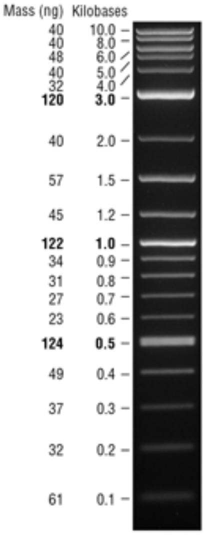

Left side of the gel has been used in Figure 2C. It represents an amelioration of RCA protocol used in the right side of the gel. Same samples were used for the two amplifications.

1: water
2: sample with cccDNA concentration 0.01 copies/cell)
3: sample with cccDNA concentration 0.11 copies/cell)
4: sample with cccDNA concentration 1.2 copies/cell)
5: sample with cccDNA concentration 10 copies/cell)
6: HBV plasmid
7: HBV plasmid
8: water
